# Supplementary material for: Decreased content of ascorbic acid (vitamin C) in the brain of knockout mouse models of Na+,K+-ATPase-related neurologic disorders
Source: PLoS One. 2021 Feb 5;16(2):e0246678. doi: 10.1371/journal.pone.0246678 (PMC7864419; doi:10.1371/journal.pone.0246678)
Supplement: Attachment — Submitted filename: Response to Reviewers 20210121.docx [file pone.0246678.s002.docx]

Response to Reviewers

January 21^th^, 2021

We would like to extend our sincere thanks to the reviewers for their careful evaluation of our manuscript. We have responded to the query raised below. We are glad to have had to opportunity to correct this sentence. Thank you very much.

Sincerely, Dr. K. Ikeda,
*on behalf of all of the authors*

Reviewer #2: All of my comments have been adequately addressed apart from my point on the ambiguity of the following passage: "Surviving 1-cell embryos were transferred into the oviducts of pseudopregnant ICR females. Slc23a2-KO (strain deposited in the RIKEN BioResource Research Center) and Slc23a2-floxed mice were generated as previously described [40,41]. Because all the latter were found to be random integrations in non-homologous chromosomes, we used only Slc23a2-KO mice for the subsequent analyses." Please clarify in the manuscript what is being referred to by "all the latter".

Thank you very much for this valuable comment. In the re-revised manuscript, we have clarified "all the latter" to “all the Slc23a2-floxed mice”.
